# EMB2/374: Evidence and not Evidence-based Products Offered for Smoking Cessation on the World Wide Web

**DOI:** 10.2196/jmir.1.suppl1.e22

**Published:** 1999-09-19

**Authors:** J Eckldorna, E Groman

**Affiliations:** ^1^Institute of Social Medicine, ViennaAustria

**Keywords:** Internet, Smoking, Cessation, Herbal, Quit

## Abstract

**Introduction:**

If people want to stop smoking, they look for advice how to do so. In recent years the internet has come up as a convenient and comprehensive means of information. However, a number of products to quit smoking is offered. We have examined smoking cessation products distributed via the internet and evaluated them from a scientific point of view. This short abstract can only provide a short insight into smoking cessation products.

**Methods:**

The study was conducted by using the search string "stop+smoking" in two of the most popular internet search engines, yahoo (www.yahoo.com) and lycos (www.lycos.com). Sites offering only advice were excluded. Only sites that offered products to stop smoking were chosen. These products were then evaluated, by screening for published papers in peer-reviewed journals. In one case we tested a product measuring resulting carbon monoxide levels.

**Results:**

Fifteen different products were identified. Eight out of the fourteen products could be ordered by internet, six are described in detail in the internet, but could only be obtained in the pharmacy. Two-third of the products, however, do not have any scientific background: Two products were filters (1+2) to reduce the nicotine supply. Other products were a CD ROM (3) with relaxing products and a pocket-safe for cigarettes (4). The main ingredients in five products were herbal extracts (5-9), that have no proven effect on nicotine addiction. Consumption of herbal cigarettes (10) resulted in high carbon monoxide values (cf. E.Groman et al. A harmful aid to stopping smoking. Lancet 9151; 353: 466-467). Just five products were based on a scientific findings (published in peer reviewed journals). These were the nicotine replacement products: patch (11), gum (12), inhaler (13), nasal spray (14) and bupropion (15).

**Discussion:**

The results show that most of the products lack a scientific background. People spend their money on untrustworthy products and get discouraged if this products do not help them. Adverse health effects after consumption of herbal cigarettes, which are sold for smoking cessation can not be excluded. This example shows the necessity of continuous assessment and observation of the market, and the need for scientific studies on smoking cessation products. An international Web site (which should be found by search-engines on a leading place) hosted by scientists could prevent misleading one-ways by routinely checking the sites. If such a control exists, unreliable methods like a safe for cigarettes, that opens at a certain time to reduce smoking (or if you take the battery out) can be shown up. Another issue is the question of actuality: Most of the homepages have been installed in 1997 and not been updated until now. Again, an international body to examine the sites would be useful.

